# Low Endogenous LH on the COS Initiation Day of a GnRH-Agonist Regimen Increases the Risk of Early Pregnancy Loss and Adverse ART Outcomes

**DOI:** 10.3389/fendo.2022.830567

**Published:** 2022-02-21

**Authors:** Xi Luo, Lei Li, Na Lin, Rui Ma, Yonggang Li, Ze Wu

**Affiliations:** ^1^ Department of Reproductive Medicine, The First People’s Hospital of Yunnan Province, Kunming, China; ^2^ Reproductive Medical Center of Yunnan Province, The Affiliated Hospital of Kunming University of Science and Technology, Kunming, China; ^3^ Faculty of Life Science and Technology, Kunming University of Science and Technology, Kunming, China; ^4^ Medical School, Kunming University of Science and Technology, Kunming, China

**Keywords:** luteinizing hormone, early pregnancy loss, live birth, pituitary suppression, gonadotropin releasing hormone agonist

## Abstract

**Objective:**

To assess the impact of serum luteinizing hormone (LH) levels on the day of initiation of controlled ovarian stimulation (COS) after pituitary suppression on early pregnancy loss and assisted reproductive technology (ART) outcomes.

**Design:**

Retrospective cohort study.

**Setting:**

University-affiliated hospital.

**Patients:**

A total of 9540 normogonadotrophic patients were treated with a GnRH agonist for *in vitro* fertilization (IVF). Based on the serum concentration of LH on the COS initiation day, patients were divided into low (<1 mIU/mL, n=2838), medium (1–1.49 mIU/mL, n=3357), or high (≥1.5 mIU/mL, n=3345) LH groups and received either fresh embryo transfer (ET) or frozen ET (women with high ovarian response, insufficient endometrial thickness, or requesting frozen ET). A total of 6279 cycles were fresh ET (1960, 2222, and 2097 in the low, medium, and high LH groups, respectively).

**Intervention(s):**

During IVF/ICSI, a GnRH agonist was used to suppress pituitary function in the midluteal phase or follicular phase, and then gonadotropin was used to induce COS.

**Main Outcome Measure(s):**

The early pregnancy loss rate (ePLR) and live-birth rate (LBR) for fresh ET, as well as the cumulative ePLR and LBR for the entire ovarian stimulation cycle, were compared.

**Results:**

In the fresh ET cycles, the high, medium and low LH groups had an ePLR of 8.6%, 11.9% and 12.5%, respectively, and LBR of 42.1%, 37.9% and 37.5%, respectively. There were no significant differences in terms of clinical pregnancy rate (CPR), late pregnancy loss rate (lPLR), and ectopic pregnancy rate (EPR) among the three LH groups. For the entire ovarian stimulation cycle, the high LH group had a greater number of retrieved oocytes compared with the low and medium LH groups. Among the groups of high, medium and low LH, the cumulative CPR were 72.8%, 69.8% and 68.8%, respectively, and the cumulative LBR were 63.4%, 60.4% and 58.5%, respectively. There were no significant differences in the cumulative ePLR, lPLR, or EPR. After multivariable logistic regression, compared with the high LH group, the adjusted odds ratio of early pregnancy loss in the low and medium LH group were 1.429 (1.065-1.919, *P* = 0.018) and 1.389 (1.041-1.853, *P* = 0.026).

**Conclusions:**

After pituitary suppression by a GnRH-agonist during IVF, a low LH level (<1.5 mIU/mL) on the COS initiation day was associated with adverse ART outcomes—including fewer oocytes, higher ePLR and lower LBR in fresh ET—and lower cumulative CPR and LBR in the entire ovarian-stimulation cycle. And LH on the COS initiation day was an independent factor affecting ePLR after multivariate regression.

## Introduction

The development of *in vitro* fertilization-embryo transfer (IVF-ET) and other assisted reproductive technologies (ART) has advanced our understanding of the role of gonadotropins in human reproductive function (1, 2). During a natural menstrual cycle, luteinizing hormone (LH) plays a key role in promoting oocyte maturation and triggering the ovulatory process in the late follicular phase (3, 4). At the stage of follicular development, LH can interact with the LH/chorionic gonadotrophin receptors (LHCGRs) on theca cells to promote the production of androgen. The androgen then disseminates into the surrounding granulosa cells and is eventually aromatized to estrogen to promote follicular development (5). However, LH may play a role in nongonadal tissue. In previous work, during the early follicular phase, functional LHCGRs were detected in human endometrium despite serum LH being at a low level, suggesting that LH might be involved in facilitating reproductive processes in the endometrium (6). However, the role of LH in the early follicular phase has not been closely studied. Therefore, it is reasonable to speculate that LH in early follicular development may be related to the outcome of ART treatment.

It is commonly believed that the low level of LH before controlled ovarian stimulation (COS) during a GnRH-agonist regimen has no effect on the outcome of ART (7–9), especially on early pregnancy loss. Moreover, in the process of COS, the effect of endogenous LH on pregnancy outcome is controversial (10). Such results may have been limited by the lack of consensus on ART. However, with the development of the ART, there was more understanding of the role of LH. In spite of this, the effect of LH level on the outcome has not been fully studied. In agonist regimens, LH concentration was usually lower than the physiological level at the initiation of COS. In addition, due to the critical role of LH in the late phase of follicular development, exogenous LH is frequently added during the COS process (11–13), which could further obscure the important role of endogenous LH in the early phase of follicular development. For the study of the role of LH in early follicular stage, the endogenous LH level on the initiation day may be more appropriate than that after COS.

In the present study, the hypothesis is that LH could have a more important role during the early stage of follicular development and that a low level of LH on the COS initiation day might compromise ART outcomes. We analyzed the effects of serum LH levels on the COS initiation day after pituitary suppression on reproductive outcome and discussed the role of LH in the follicular phase on the preparatory reproductive process.

## Materials and Methods

### Study Design and Patient Selection

This was a retrospective cohort study performed at the Affiliated Hospital of Kunming University of Science and Technology, Kunming, China, with approval from the Ethics Committee of the First People’s Hospital of Yunnan Province (number: KHLL2020-KY013). Data were collected from patients who visited our hospital from January 1, 2015, to December 31, 2018. Data were de-identified to ensure patient privacy.

To assess the impact of LH on the general population of women, the inclusion criteria for patient enrollment included the following: 1), Normogonadotrophic patients with basal FSH less than 10 mIU/mL on the second day of menstruation received their first cycle of IVF-ET treatment; 2), the pituitary suppression protocol included a long-acting GnRH-agonist; and 3), pituitary suppression was only applied starting at the mid-luteal phase or the early follicular phase. Exclusion criteria included endometriosis, polycystic ovary syndrome (PCOS), family genetic diseases or karyotype abnormalities, immune factors, female reproductive tract malformations, uterine fibroids, no embryo transfer occurred (no oocytes retrieved or no available embryos, available embryos but none transferred), and serious bleeding or complications.

### COS and IVF-ET Protocols

All patients were treated with a GnRH agonist protocol that has been used and described previously (14). Briefly, the long-acting GnRH-agonist triptorelin (Diphereline, Ipsen, Signes, France) was used on the second day of the menstruation or in the middle of the luteal phase (3.75 mg administered for the follicular phase and 1.25 mg for the luteal phase). The dosage of agonist was accordance with the guideline. When pituitary suppression was completed after 14–21 days, serum FSH, LH, E2, and progesterone levels were evaluated. The endometrial thickness was measured by ultrasonography. When the patient had E2 < 50 pg/mL, endometrial thickness < 5 mm, and absence of cysts, a fixed dose of 150–225 IU of exogenous gonadotropin (Gn) (rFSH, Gonal-F, Merck-Serono, Aubonne, Switzerland) was administrated for ovarian stimulation. Ultrasonography was used to monitor follicular growth during the COS process. Based on the patient’s reproductive status, the amount of Gn was adjusted appropriately. When the largest diameter of 2 dominant follicles was ≥ 18 mm, serum levels of LH, E2, and P were determined and 5000 IU of exogenous hCG was injected on the same day. This was followed by ultrasound-guided oocyte retrieval 34–36 hours later. All serum hormone tests were completed using a UniCel DxI 800 Access Immunoassay System (Beckman-Coulter, Brea, CA, USA).

Conventional IVF or ICSI was selected according to male semen assessments. Pronuclear formation of the zygote was observed on the first day after oocyte retrieval to evaluate the normal fertilization rate. The number of available embryos and the proportion of high-quality (defined with 2PN zygote on the 1^st^ day, 6-8 blastomeres on 3^rd^ day, and cell debris less than 20%) embryos were evaluated. When the number of available embryos was > 6, blastocyst culture and fresh-cleavage embryo transfer were performed. However, for patients with high ovarian response or insufficient endometrial thickness, or who actively requested frozen embryo transfer (FET), fresh embryo transfer (ET) was canceled, and the cleavage embryos were frozen, and FET was performed in the follow-up cycle. Luteal support was initiated immediately after oocyte retrieval. A biochemical pregnancy test was performed on the 14^th^ days after ET. If the biochemical pregnancy test was positive, luteal support was sustained until the 8^th^ weekend.

### Outcome Measurements

Patient cycle information was retrieved through review of medical records. Based on the serum concentration of LH on the COS initiation day and tertile of population distribution, patients were divided into 3 groups: low LH (<1 mIU/mL), medium LH (1–1.49 mIU/mL), and high LH (≥1.5 mIU/mL). Primary outcomes were early pregnancy loss rate (ePLR) and live-birth rate (LBR); and secondary outcomes were clinical pregnancy rate (CPR), late pregnancy loss rate (lPLR) and ectopic pregnancy rate (EPR). Biochemical pregnancy was defined by serum β-hCG test positive, and clinical pregnancy was defined by presenting gestational sac using ultrasound scan after 6 weeks of ET. A miscarriage within the first trimester was defined as early pregnancy loss, and a miscarriage after 12 weeks was defined as late pregnancy loss. The PLR refers to the ratio of the number of pregnancy loss cycles to the number of clinical pregnancy cycles, and the CPR, EPR and LBR refers to ratio of corresponding cycles to the number of transfer cycles. The cumulative rate was defined as the proportion of an event that occurred at least once during the entire ovarian stimulation cycle including all fresh and frozen transfer cycles.

### Statistical Methods

All statistical analyses were conducted in SPSS (version 26.0, Armonk, NY, USA). A *P* < 0.05 in a 2-tailed test was considered statistically significant. We compared the CPR, ePLR, lPLR, EPR, and LBR in three LH groups. For continuous variables, The Shapiro-Wilk’s test combined with P-P plots was used to evaluate normality. Normally distributed data are presented as mean ± SD and were compared using one-way ANOVA. Non-normally distributed data are presented as median and interquartile range (25^th^–75^th^ percentiles) and were compared by the Kruskal-Wallis test. The Bonferroni method was used to correct the *P* value for multiple comparisons. The categorical variables were analyzed by the Chi-squared test, with the *P* value for multiple comparison adjusted using the Bonferroni method.

For the multivariate logistic regression, continuous variables, such as age, BMI, serum hormone concentration, endometrial thickness, and number of oocytes or embryos, were transformed into categorical variables based on recognized cut-off values and data distribution. All variables were included for analysis to create the final regression model using the forward likelihood ratio (LR) method. To analyze the correlation between LH on the initiation day and on the hCG trigger day, simple linear regression was used, and Pearson R^2^ and the slope of the regression line equation were calculated. Odds ratios (OR) with 95% confidence intervals (CI) were reported.

## Results

**Figure 1:**
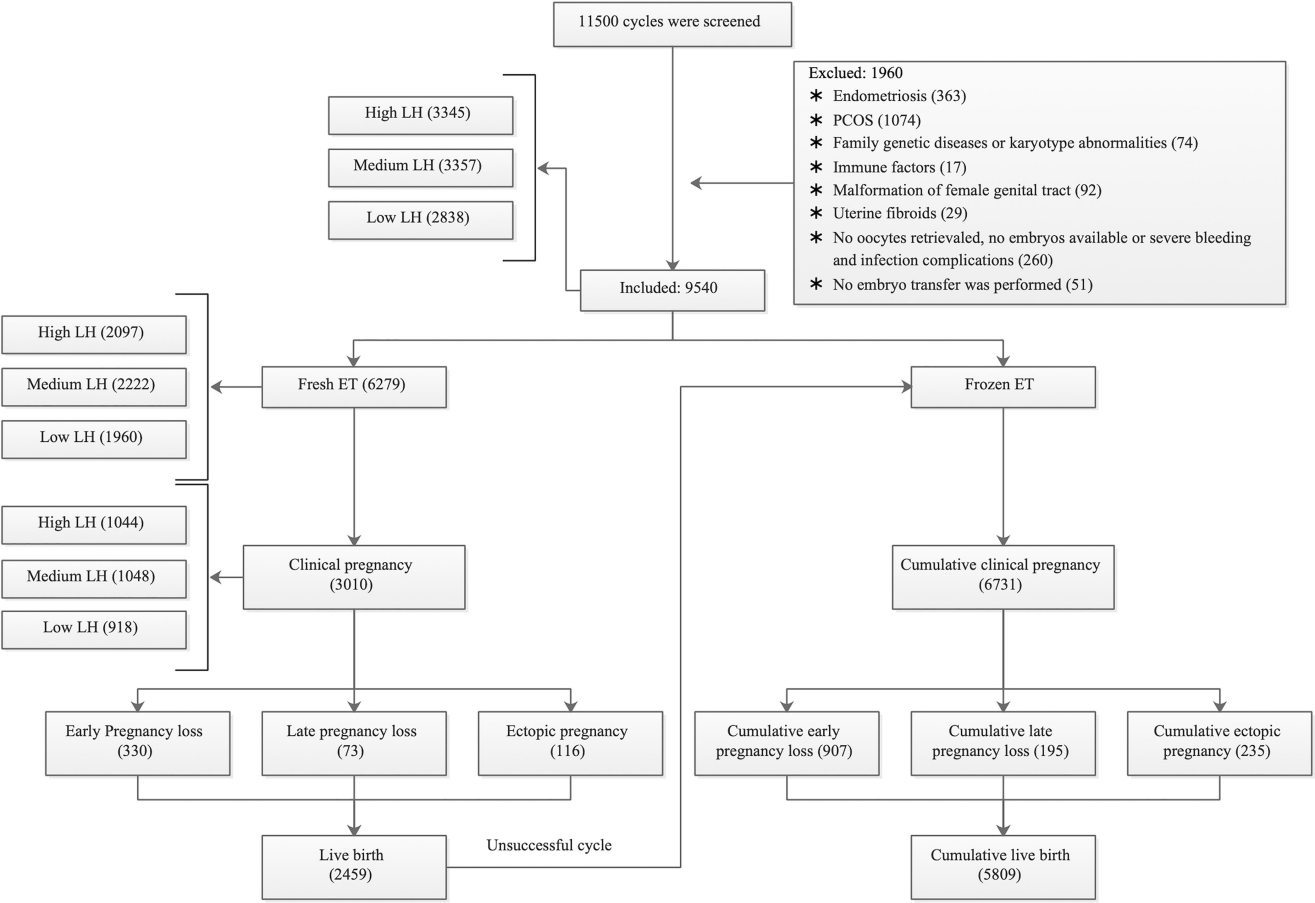
Flow chart of patient and cycle selection. PCOS = polycystic ovary syndrome; ET = embryo transfer. According to the LH level on the COS initiation day, we grouped high LH as ≥1.50 mIU/ml, medium LH as ranging from 1.00–1.49 mIU/ml, and low LH as <1.00 mIU/ml.

We ultimately included 9,540 cycles (**Figure 1**). Based on the serum concentration of LH on the COS initiation day, there were 2838 cycles in the low LH group, 3357 cycles in the medium LH group, and 3345 cycles in the high LH group. A total of 6279 cycles underwent fresh ET, with 1960, 2222, and 2097 cycles in the low, medium, and high LH groups, respectively.

### Baseline Analysis of LH Groups on the Day of Initiation in the Entire Stimulation Cycle

**Table 1** summarizes the clinical baseline data from the 3 levels of LH on the day of initiation. The number of oocytes retrieved, as well as the number of embryos available for ET in the low and medium LH groups, were significantly lower than those in the high LH group. There was no significant difference among the 3 LH groups with regards to the normal fertilization rate or the high-quality embryo rate. The high LH group had a significantly greater number of embryos available for ET than the low and medium LH groups. Although the median (25^th^–75^th^ percentiles) of some variables was similar, their *P* value was still <0.05 due to the difference in distribution of large sample size. All variables were ultimately included in the subsequent multivariate binary logistic regression analysis.

**Table 1:** Baseline analysis of LH groups on the COS initiation day.

Characteristics	Low LH (n=2838) <1 mIU/mL	Medium LH (n=3357) 1–1.49 mIU/mL	High LH (n=3345) ≥1.5 mIU/mL	*P* value
Age (years)	30.62 ± 4.06^a^	30.21 ± 3.95^b^	29.93 ± 3.96^c^	<0.001
BMI	21.82 ± 2.77	21.77 ± 2.67	21.80 ± 2.67	0.753
Duration of infertility (years)	4 (2–6)^a^	3 (2–6)^a,b^	3 (2–5)^b^	0.036
Primary infertility, n (%)	1242 (43.8%)^a^	1541 (45.9%)^a,b^	1569 (46.9%)^b^	0.043
Gravidity	1 (0–1)	1 (0–1)	1 (0–1)	0.056
Parturition history, n (%)	262 (9.2%)^a^	204 (6.1%)^b^	190 (5.7%)^b^	<0.001
Vaginal birth, n (%)	180 (6.3%)^a^	154 (4.6%)^b^	142 (4.2%)^b^	<0.001
Cesarean section births, n (%)	82 (2.9%)^a^	50 (1.5%)^b^	48 (1.4%)^b^	
Ectopic pregnancy, n (%)	595 (21.0%)^a,b^	655 (19.5%)^b^	775 (23.2%)^a^	0.001
Spontaneous abortion, n (%)	185 (6.5%)^a,b^	244 (7.3%)^b^	183 (5.5%)^a^	0.011
Non-spontaneous abortion, n (%)	481 (16.9%)	604 (18.0%)	554 (16.6%)	0.278
Causes of infertility				
Male factor	433 (15.3%)	498 (14.8%)	494 (14.8%)	0.805
Female factor	2066 (72.8%)	2476 (73.8%)	2440 (72.9%)	
Couple factor	339 (11.9%)	383 (11.4%)	411 (12.3%)	
Etiology of female infertility				
Fallopian tube factor	2074 (86.2%)^a^	2519 (88.1%)^a,b^	2528 (88.7%)^b^	0.100
Ovulation disorder	96 (4.0%)	113 (4.0%)	112 (3.9%)	
Unexplained infertility	153 (6.4%)^a^	150 (5.2%)^a,b^	135 (4.7%)^b^	
Multiple factors	82 (3.4%)	77 (2.7%)	76 (2.7%)	
Pituitary suppression regimen				
Mid-luteal phase regimen, n (%)	2702 (95.2%)^a^	3266 (97.3%)^b^	3124 (93.4%)^c^	<0.001
Early-follicular phase regimen, n (%)	136 (4.8%)^a^	91 (2.7%)^b^	221 (6.6%)^c^	
Serum hormone concentration on the day of COS initiation				
LH (mIU/mL)	0.8 (0.67–0.90)^a^	1.21 (1.11–1.34)^b^	1.90 (1.67–2.34)^c^	<0.001
FSH (mIU/mL)	1.94 (1.36–2.84)^a^	2.03 (1.49–2.85)^b^	2.24 (1.66–3.17)^c^	<0.001
E2 (pg/mL)	19 (15–25)^a^	19 (15–25)^a^	20 (15–26)^b^	<0.001
Progesterone (ng/mL)	0.55 (0.35–0.83)^a^	0.62 (0.40–0.94)^b^	0.66 (0.42–1.01)^c^	<0.001
Duration of ovarian stimulation (days)	11 (11–13)	11 (11–12)	11 (11–12)	0.097
Dosage used in ovarian stimulation (IU)	2700 (2050–3425)^a^	2575 (1950–3200)^b^	2475 (1900–3175)^c^	<0.001
Serum hormone concentration on the day of the hCG trigger				
LH (mIU/mL)	0.50 (0.37–0.67)^a^	0.66 (0.50–0.86)^b^	0.87 (0.66–1.16)^c^	<0.001
E2 (pg/mL)	3066 (1926–4321)^a^	3346 (2177–4558)^b^	3732 (2493–4855)^c^	<0.001
Progesterone (ng/mL)	1.01 (0.70–1.44)^a^	1.08 (0.76–1.54)^b^	1.12 (0.78–1.60)^b^	<0.001
Endometrial thickness on the day of the hCG trigger (mm)	11 (10–13)^a^	12 (10–13)^a,b^	12 (10–13)^b^	0.003
Number of oocytes retrieved	13 (9–18)^a^	14 (10–19)^b^	14 (10–20)^c^	<0.001
Methods of fertilization				
Conventional IVF, n (%)	2233 (78.7%)	2658 (79.2%)	2696 (80.6%)	0.146
ICSI, n (%)	605 (21.3%)	699 (20.8%)	649 (19.4%)	
Normal fertilization rate	80.0% (66.7%–90.5%)	80.0% (66.7%–90.0%)	80.0% (66.7%–90.0%)	0.555
High-quality embryo rate	50.0% (33.3%–70.0%)	50.0% (33.3%–71.4%)	54.5% (33.3%–71.4%)	0.069

BMI, body mass index; LH, luteinizing hormone; FSH, follicle-stimulating hormone; E2, estradiol; COS, controlled ovarian stimulation; hCG, human chorionic gonadotrophin.

Different superscript letters (a, b, c) denote significant differences in subset Pairwise comparison of 3 LH levels (P < 0.05 with Bonferroni correction).

### Comparison of Clinical Outcomes of 3 LH Groups on the Initiation Day

We compared the clinical outcome data of fresh ET and the entire stimulation cycle (**Table 2**). The results indicated that in the fresh ET cycle, the ePLRs in the low and medium LH groups were significantly higher than in the high LH group, and the LBRs in these groups were lower than in the high LH group. However, there was no difference in the ePLR and the LBR between the low and medium LH groups. For the entire ovarian stimulation cycle, a higher LH resulted in a higher rate of fresh ET cancelation, and the cumulative CPR and LBR of the low and medium LH groups were significantly lower than in the high LH group. All rates were calculated based on the clinical pregnancy.

**Table 2:** Clinical outcomes of LH groups on COS initiation day.

Characteristics	Low LH <1 mIU/mL	Medium LH 1–1.49 mIU/mL	High LH ≥1.5 mIU/mL	*P* value
Number of fresh embryos transfer cycles	1960 (69.1%)^a^	2222 (66.2%)^b^	2097 (62.7%)^c^	<0.001
Number of single-embryo transfer cycles	216 (11.0%)	228 (10.3%)	204 (9.7%)	0.398
Number of cycles with at least 1 high-quality embryo transfer	1678 (85.6%)^a^	1923 (86.5%)^a,b^	1858 (88.6%)^b^	0.015
Biochemical pregnancy	1001 (51.1%)	1136 (51.1%)	1129 (53.8%)	0.123
Clinical pregnancy	918 (46.8%)	1048 (47.2%)	1044 (49.8%)	0.113
Early pregnancy loss	115 (12.5%)^a^	125 (11.9%)^a^	90 (8.6%)^b^	0.010
Late pregnancy loss	28 (3.1%)	22 (2.1%)	23 (2.2%)	0.332
Ectopic pregnancy	33 (3.6%)	44 (4.2%)	39 (3.7%)	0.763
Live births	735 (37.5%)^a^	842 (37.9%)^a^	882 (42.1%)^b^	0.004
Whole ovarian-stimulation cycles	2838	3357	3345	–
Number of embryos available	4 (3–6)^a^	4 (3–6)^b^	5 (3–7)^c^	<0.001
Cumulative clinical pregnancies	1953 (68.8%)^a^	2343 (69.8%)^a^	2435 (72.8%)^b^	0.001
Cumulative early pregnancy loss	283 (14.5%)	313 (13.4%)	311 (12.8%)	0.248
Cumulative late pregnancy loss	56 (2.9%)	65 (2.8%)	74 (3.0%)	0.858
Cumulative ectopic pregnancy	66 (3.4%)	79 (3.4%)	90 (3.7%)	0.789
Cumulative live births	1659 (58.5%)^a^	2028 (60.4%)^a^	2122 (63.4%)^b^	<0.001

Different superscript letters (a, b, c) denote significant differences in subset Pairwise comparison of 3 LH levels (P < 0.05 with Bonferroni correction).

### Factors Affecting Early Pregnancy Loss and Live Births in Fresh ET Cycles

In the multivariate regression model, the main variables affecting early pregnancy loss after fresh ET included age, LH on the day of COS initiation, endometrial thickness on the hCG trigger day, and the number of transferred embryos (**Table 3**). The model included 3010 clinical pregnancy-positive cycles, 330 of which exhibited early pregnancy loss. Compared with the high LH group, the crude ORs of the low LH and the medium LH groups were 1.518 (95% CI 1.134–2.032, *P* = 0.005) and 1.436 (95% CI 1.079–1.910, *P* = 0.013), respectively, with a *P* value for the trend of 0.011. The adjusted ORs were 1.429 (95% CI 1.065–1.919, *P* = 0.018) and 1.389 (95% CI 1.041–1.853, *P* = 0.026), respectively; and the *P* value for the trend was 0.033. These results indicated that low LH on the COS initiation day might lead to a higher ePLR.

**Table 3:** Univariate and multivariate analyses of early pregnancy loss and live births in fresh ET cycles.

	n	Crude OR	95% CI	P value	Adjusted OR	95% CI	P value
**Early pregnancy loss regression** **^a^**							
Age (years)							
<35 (Ref)	2626	–	–	–	–	–	–
35–39	367	2.096	1.564–2.810	<0.001	2.026	1.508–2.722	<0.001
≥40	17	3.841	1.342–10.989	0.012	3.553	1.232–10.244	0.019
LH on the day of the COS initiation day (mIU/mL)							
≥1.5 (Ref)	1044	–	–	–	–	–	–
<1	918	1.518	1.134–2.032	0.005	1.429	1.065–1.919	0.018
1–1.49	1048	1.436	1.079–1.910	0.013	1.389	1.041–1.853	0.026
Endometrial thickness on the day of the hCG trigger (mm)							
<10 (Ref)	408	–	–	–	–	–	–
10–12	1507	0.657	0.478–0.903	0.010	0.652	0.472–0.900	0.009
≥13	1095	0.655	0.468–0.915	0.013	0.663	0.473–0.930	0.017
Number of fresh transferred embryos							
1 (Ref)	183	–	–	–	–	–	–
2	2827	0.580	0.387–0.869	0.008	0.619	0.411–0.933	0.022
**Live-birth regression** **^b^**							
Age (years)							
< 35 (Ref)	5263	–	–	–	–	–	–
35–39	949	0.547	0.470–0.636	<0.001	0.592	0.506–0.693	<0.001
≥40	67	0.277	0.145–0.530	<0.001	0.292	0.151–0.566	<0.001
BMI							
≥25 (Ref)	1004	–	–	–	–	–	–
< 18.5	624	1.529	1.245–1.877	<0.001	1.426	1.153–1.765	0.001
18.5–24.9	4651	1.284	1.113–1.482	0.001	1.245	1.074–1.445	0.004
Cause of infertility							
Primary infertility (Ref)	2775	–	–	–	–	–	–
Secondary infertility	3504	1.016	0.917–1.125	0.765	1.164	1.045–1.296	0.006
Number of available embryos for ET							
<4 (Ref)	2457	–	–	–	–	–	–
4–6	2961	1.517	1.357–1.695	<0.001	1.292	1.147–1.454	<0.001
≥7	861	1.701	1.452–1.993	<0.001	1.409	1.192–1.664	<0.001
LH on the day of the hCG trigger (mIU/mL)							
< 0.5 (Ref)	1483	–	–	–	–	–	–
0.5–0.79	2432	1.288	1.125–1.475	<0.001	1.234	1.074–1.419	0.003
≥0.8	2364	1.536	1.342–1.759	<0.001	1.476	1.284–1.697	<0.001
Progesterone on the day of the hCG trigger (ng/mL)							
≥1.3 (Ref)	1738	–	–	–	–	–	–
< 0.8	2198	1.283	1.127–1.461	<0.001	1.363	1.191–1.56	<0.001
0.8–1.29	2343	1.241	1.092–1.412	0.001	1.300	1.138–1.484	<0.001
Endometrial thickness on the day of the hCG trigger (mm)							
<10 (Ref)	1042	–	–	–	–	–	–
10–12	3230	1.503	1.292–1.748	<0.001	1.489	1.276–1.739	<0.001
≥13	2007	1.999	1.704–2.345	<0.001	1.980	1.679–2.334	<0.001
Number of embryos transferred							
1 (Ref)	648	–	–	–	–	–	–
2	5631	2.566	2.111–3.118	<0.001	1.984	1.615–2.437	<0.001
Transfer of at least 1 high-quality embryo							
No (Ref)	820			–			–
Yes	5459	1.923	1.632–2.266	<0.001	1.578	1.326–1.877	<0.001
LH on the day of the COS initiation (mIU/mL)							
≥1.5 (Ref)	2097	–	–	–	–	–	–
<1	1960	0.827	0.729–0.938	0.003	–	–	0.259
1–1.49	2222	0.841	0.744–0.949	0.005	–	–	0.145

a Adjusted by age, LH on the day of COS initiation; endometrial thickness on the day of the hCG trigger; and the number of fresh transferred embryos.

b Adjusted by age, BMI, cause of infertility, LH and progesterone concentrations; endometrial thickness on the day of the hCG trigger; number of embryos transferred; transfer of at least 1 high-quality embryo; and number of available embryos for ET.

OR, odds ratio; CI, confidence interval; BMI, body mass index; LH, luteinizing hormone; hCG, human chorionic gonadotrophin.

In terms of live-birth analysis for fresh ET, a total of 6279 fresh ET cycles were included, of which 2459 cycles resulted in live births. The univariate results of LH on the day of COS initiation showed that in comparison to the high LH group, the ORs of live birth in the low and the medium LH groups were 0.827 (95% CI 0.729–0.938, *P* = 0.003) and 0.841 (95% CI 0.744–0.949, *P* = 0.005), respectively, with a *P* value for the trend of 0.004. However, in the multivariate model (**Table 3**). The LH level on the initiation day was excluded from the adjusted model and replaced with LH on the day of hCG trigger, which may be more likely to affect the final outcome of live birth. Thus, with respect to LH on the day of the hCG trigger, for the < 0.5 mIU/mL group, the crude ORs of the 0.5–0.79 mIU/mL and ≥0.8 mIU/mL groups were 1.288 (95% CI 1.125–1.475, *P* < 0.001) and 1.536 (95% CI 1.342–1.759, *P* < 0.001), respectively, with a *P* value for the trend of < 0.001. The adjusted ORs were 1.234 (95% CI 1.074–1.419, *P* = 0.003) and 1.476 (95% CI 1.284–1.697, *P* < 0.001), respectively, with a *P* value for the trend of < 0.001. The analysis results showed that on the day of hCG trigger of GnRH-agonist regimen, a higher serum concentration of LH was beneficial for live birth. The LH level on the day of COS initiation was not an independent factor that affected live births from fresh ET.

### Relationship Between the COS Initiation Day and the hCG Trigger Day With Respect to LH Concentration

**Figure 2:**
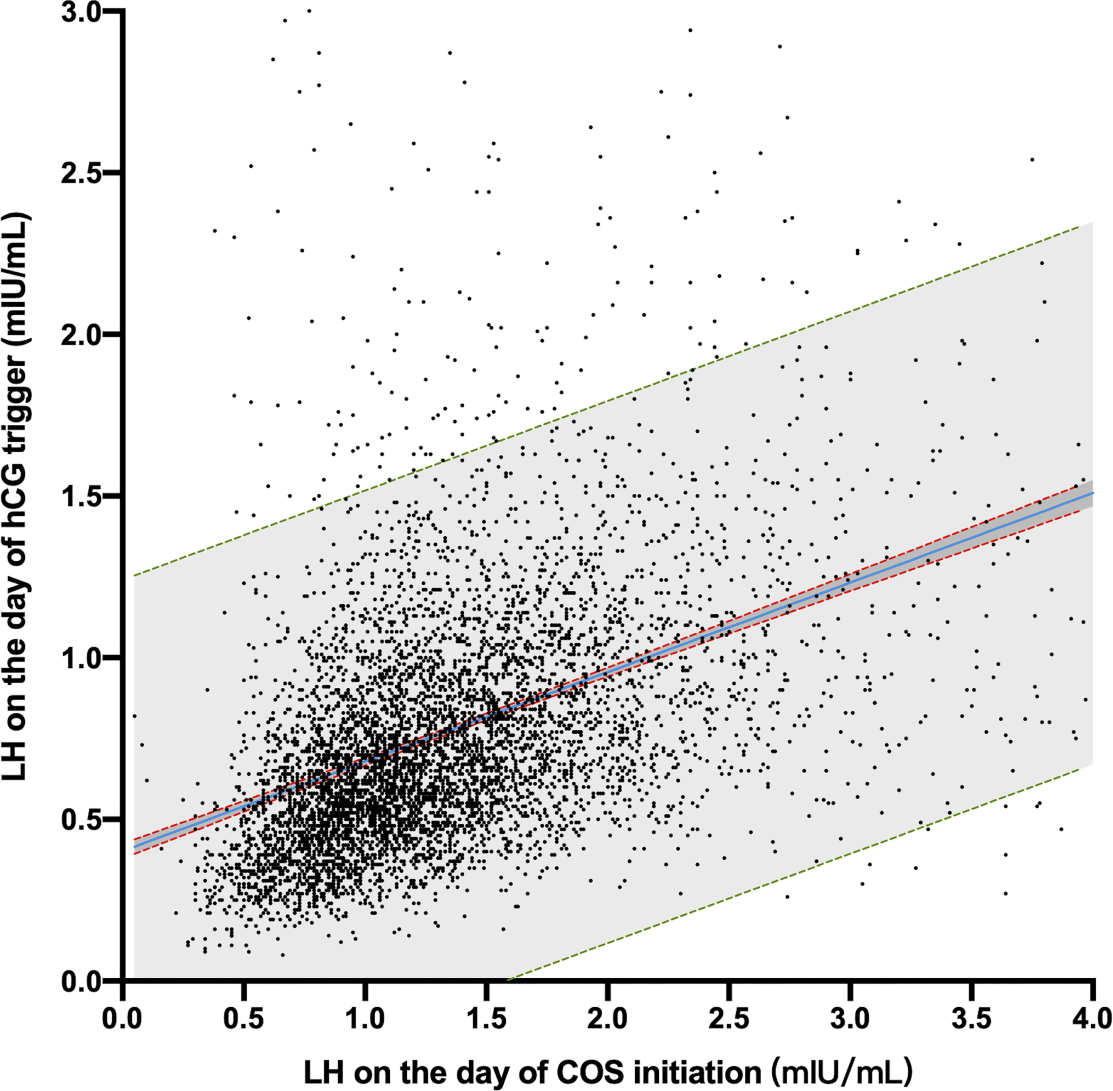
Scatter plot of LH concentrations on the COS initiation day and hCG trigger day. The blue line is the linear regression equation line. The red dotted lines represent the 95% confidence bands of the best-fit equation. The green dotted line is the 90% prediction band of the best-fit equation.

There was a linear correlation in the LH concentration between the initiation day and the hCG trigger day (Pearson R^2^ = 0.174, *P* < 0.001; with a slope of 0.277, 95% CI 0.262–0.292) (**Figure 2**), which implied that different fresh ET LBR in 3 initiation day LH groups might be caused by affecting the LH level on the day of hCG trigger.

### Subgroup Analysis of LH on the Initiation Day and Early Pregnancy Loss After Fresh ET

To exclude the influence of advanced age, obesity, history of miscarriage, nulligravidity, poor ovarian function, and low-quality embryos, we performed a subgroup multivariate regression analysis on the fresh ET cycles (**Table 4**). The results indicated that LH on the day of COS initiation was significantly associated with early pregnancy loss in women of non-advanced age (< 35 years of age), BMI < 25, no history of miscarriage and artificial abortion, history of gravidity, normal ovarian reserve (AFC >5, number of retrieved oocytes >3), and those who experienced the transfer of at least one high-quality embryo or of two embryos. Compared with the high-LH group, the adjusted OR ranges for the low and medium LH groups were 1.404–1.567 and 1.357–1.639, respectively. These results confirmed that the ePLR in the low and medium LH groups was still significantly higher than that in the high LH group in patients with a better reproductive history.

**Table 4:** Subgroup analysis of LH in early pregnancy loss in fresh ET cycles.

LH level (mIU/mL)	n	Adjusted OR	95% CI	*P* value
Age <35				
≥1.5 (Ref)	944	–	–	–
< 1	774	1.518	1.094–2.105	0.012
1–1.49	908	1.422	1.033–1.958	0.031
BMI<25				
≥1.5 (Ref)	889	–	–	–
< 1	784	1.426	1.031–1.971	0.032
1–1.49	905	1.439	1.051–1.970	0.023
No history of miscarriage or artificial abortion				
≥1.5 (Ref)	813	–	–	–
< 1	710	1.567	1.116–2.200	0.009
1–1.49	773	1.357	0.964–1.911	0.081
History of gravidity				
≥1.5 (Ref)	548	–	–	–
< 1	497	1.404	0.942–2.093	0.096
1–1.49	553	1.639	1.118–2.402	0.011
AFC ≥ 6				
≥1.5 (Ref)	1025	–	–	–
< 1	902	1.456	1.082–1.959	0.013
1–1.49	1033	1.385	1.035–1.853	0.029
Number of oocytes retrieved ≥4				
≥1.5 (Ref)	1028	–	–	–
< 1	896	1.489	1.104–2.008	0.009
1–1.49	1023	1.418	1.057–1.902	0.020
Transfer of at least 1 high-quality embryo				
≥1.5 (Ref)	966	–	–	–
< 1	812	1.470	1.078–2.005	0.015
1–1.49	959	1.357	1.002–1.838	0.048
Transfer of 2 embryos				
≥1.5 (Ref)	982	–	–	–
< 1	861	1.506	1.105–2.053	0.010
1–1.49	984	1.449	1.069–1.964	0.017

Adjusted by age, LH on the day of COS initiation, endometrial thickness on the day of the hCG trigger, and the number of fresh transferred embryos.

OR, odds ratio; CI, confidence interval; BMI, body mass index; AFC, antral follicle count; COS, controlled ovarian stimulation; hCG, human chorionic gonadotrophin.

### Factors Affecting Cumulative Clinical Pregnancy and Live-Birth Rates

Since different LH levels on the day of initiation of COS correlated with different cumulative CPR and LBR, we analyzed the relationship between LH and them (**Table 5**). The regression model for clinical pregnancy included 9540 cycles and 6731 cycles of cumulative clinical pregnancy. Univariate analysis showed that with respect to cumulative live births and compared with the high-LH group, the ORs for the low and medium LH groups were 0.825 (95% CI 0.739–0.921, *P* =0.001) and 0.864 (95% CI 0.777–0.960, *P* = 0.007), respectively. The regression model for live births included 9540 cycles, and there were 5809 cycles of cumulative clinical pregnancies. Univariate analysis showed that compared with the high LH group in cumulative live births, the ORs for the low and medium LH groups were 0.811 (95% CI 0.732–0.899, *P <*0.001) and 0.879 (95% CI 0.797–0.971, *P* = 0.011), respectively. However, multivariate regression results suggested that LH on the initiation day was not an independent variable affecting cumulative clinical pregnancy and live birth.

**Table 5:** Univariate and multivariate analyses of clinical pregnancies and live births in entire ovarian cycles.

	n	Crude OR	95% CI	P value	Adjusted OR	95% CI	P value
**Cumulative pregnancy regression** **^a^**							
Age (years)							
<35 (Ref)	8080	–	–	–	–	–	–
35–39	1360	0.582	0.517–0.656	<0.001	0.718	0.627–0.822	<0.001
≥40	100	0.262	0.176–0.392	<0.001	0.306	0.198–0.473	<0.001
BMI							
≥25 (Ref)	1379	–	–	–	–	–	–
<18.5	985	1.342	1.122–1.604	0.001	1.224	1.003–1.494	0.046
18.5–24.9	7176	1.280	1.132–1.447	<0.001	1.233	1.075–1.413	0.003
Dosage of Gn (IU)							
≥3000 (Ref)	3082	–	–	–	–	–	–
<2100	2812	2.385	2.124–2.677	<0.001	1.192	1.034–1.374	0.015
2100–2999	3646	1.706	1.539–1.890	<0.001	1.138	1.012–1.280	0.031
Progesterone on the day of the hCG trigger (ng/mL)							
≥1.3 (Ref)	3456	–	–	–	–	–	–
<0.8	2721	0.931	0.834–1.039	0.201	1.265	1.113–1.437	<0.001
0.8–1.29	3363	1.029	0.927–1.143	0.592	1.219	1.083–1.372	0.001
Endometrial thickness on the day of the hCG trigger (mm)							
<10 (Ref)	1489	–	–	–	–	–	–
10–12	4872	1.384	1.225–1.563	<0.001	1.401	1.224–1.604	<0.001
≥13	3179	1.746	1.530–1.993	<0.001	1.847	1.594–2.139	<0.001
Number of oocytes retrieved							
<4 (Ref)	191	–	–	–	–	–	–
4–9	2110	2.380	1.747–3.243	<0.001	1.717	1.242–2.374	0.001
10–19	4968	4.985	3.680–6.754	<0.001	2.355	1.697–3.268	<0.001
≥20	2271	8.228	6.002–11.282	<0.001	2.575	1.805–3.673	<0.001
Total number of available embryos for ET							
<4 (Ref)	3088	–	–	–	–	–	–
4–6	4182	3.644	3.295–4.029	<0.001	2.823	2.525–3.157	<0.001
≥7	2270	11.203	9.547–13.147	<0.001	7.185	5.972–8.645	<0.001
LH on the day of initiation (mIU/mL)							
≥1.5 (Ref)	3345	–	–	–	–	–	–
<1	2838	0.825	0.739–0.921	0.001	–	–	0.679
1–1.49	3357	0.864	0.777–0.960	0.007	–	–	0.250
**Cumulative live-birth regression** **^b^**							
Age (years)							
< 35 (Ref)	8080	–	–	–	–	–	–
35–39	1360	0.484	0.431–0.543	<0.001	0.574	0.505–0.654	<0.001
≥40	100	0.210	0.135–0.327	<0.001	0.252	0.158–0.402	<0.001
BMI							
≥25 (Ref)	1379	–	–	<0.001	–	–	0.014
< 18.5	985	1.388	1.174–1.640	<0.001	1.161	0.961–1.402	0.122
18.5–24.9	7176	1.318	1.173–1.480	<0.001	1.212	1.065–1.381	0.004
FSH on the day of initiation (mIU/mL)							
<1.6 (Ref)	2772	–	–	–	–	–	–
1.6–2.59	3580	0.875	0.789–0.970	0.011	0.904	0.808–1.012	0.081
≥2.6	3188	0.712	0.641–0.791	<0.001	0.824	0.732–0.928	0.001
Dosage of Gn (IU)							
≥3000 (Ref)	3082	–	–	–	–	–	–
<2100	2812	2.213	1.989–2.462	<0.001	1.138	0.998–1.298	0.054
2100–2999	3646	1.673	1.518–1.845	<0.001	1.142	1.022–1.276	0.019
LH on the day of the hCG trigger (mIU/mL)							
< 0.5 (Ref)	2448	–	–	–	–	–	–
0.5–0.79	3657	1.095	0.987–1.216	0.088	1.083	0.966–1.215	0.171
≥0.8	3435	1.132	1.018–1.259	0.022	1.174	1.043–1.321	0.008
Progesterone on the day of the hCG trigger (ng/mL)							
≥1.3 (Ref)	3456	–	–	–	–	–	–
< 0.8	2721	0.947	0.855–1.050	0.301	1.226	1.088–1.380	0.001
0.8–1.29	3363	1.025	0.930–1.130	0.618	1.166	1.046–1.300	0.005
Endometrial thickness on the day of the hCG trigger (mm)							
<10 (Ref)	1489	–	–	–	–	–	–
10–12	4872	1.413	1.257–1.588	<0.001	1.413	1.243–1.605	<0.001
≥13	3179	1.727	1.524–1.957	<0.001	1.768	1.542–2.028	<0.001
Number of oocytes retrieved							
<4 (Ref)	191	–	–	–	–	–	–
4–9	2110	2.226	1.606–3.084	<0.001	1.572	1.118–2.209	0.009
10–19	4968	4.377	3.178–6.028	<0.001	2.079	1.475–2.931	<0.001
≥20	2271	6.368	4.588–8.839	<0.001	2.058	1.428–2.966	<0.001
Number of available embryos for ET							
<4 (Ref)	3088	–	–	–	–	–	–
4–6	4182	3.033	2.754–3.340	<0.001	2.452	2.202–2.731	<0.001
≥7	2270	7.584	6.656–8.642	<0.001	5.381	4.598–6.299	<0.001
LH on the day of initiation (mIU/mL)							
≥1.5 (Ref)	3345	–	–	–	–	–	–
<1	2838	0.811	0.732–0.899	<0.001	–	–	0.759
1–1.49	3357	0.879	0.797–0.971	0.011	–	–	0.495

a Adjusted by age, BMI, history of ectopic pregnancy, history of non-spontaneous abortion, dosage of Gn, progesterone and endometrial thickness on the day of hCG trigger, number of oocytes retrieved, normal fertilization rate, high-quality embryo rate, and the total number of available embryos for ET.

b Adjusted by age, BMI, history of ectopic pregnancy, FSH on the day of initiation, progesterone and LH on the day of the hCG trigger, endometrial thickness on the day of the hCG trigger, number of oocytes retrieved, normal fertilization rate, high-quality embryo rate, and the total number of available embryos for ET.

OR, odds ratio; CI, confidence interval; LH, luteinizing hormone; COS, controlled ovarian stimulation; hCG, human chorionic gonadotrophin; BMI, body mass index; FSH, follicle-stimulating hormone; E2, estradiol.

## Discussion

The results of 9540 GnRH-agonist cycles and 6279 fresh ET cycles implied that low LH concentration (<1.5 mIU/ml) on the day of COS initiation elevated the ePLR and reduced the LBR after fresh ET, as well as the cumulative CPR and LBR in the entire stimulation cycle. Furthermore, regression analysis also revealed that the LH level on the COS initiation day was an independent factor affecting the ePLR, and on the hCG trigger day was affecting the LBR in fresh ET. In addition, for women of non-advanced age, normal ovarian function, and a thicker endometrium on the day of the hCG trigger, a low LH level also led to an elevation in the ePLR, contradicting the results from Westergaard et al. (7), Bjercke et al. (8), and Cabrera et al. (9), which concluded that the serum LH level on the day of COS initiation was not correlated with ePLR.

More than a decade ago, there was no relatively unified consensus on ART technology, and the methods of COS and follow-up support were quite different among the centers. With the advancement of technology, the current clinical outcome has been significantly improved. In the study by Westergaard et al., The CPR was only about 32.9% (7). Likewise, in the study by Bjercke et al., the ePLR was nearly 28% (8) In our study, the overall clinical pregnancy rate is 47.9%, and in the clinical pregnancy positive cycle, the ePLR is only 11.0%. These improvements are because we can deal with the known risk factors according to the existing consensus and experience. Poor prognosis may obscure the role of LH in the determination of the ART outcome. Second, early pregnancy loss is a small-probability event. In a statistical analysis with a small sample size, a type-II statistical error is prone to occur (15), which may lead to false-negative results. In addition, in the study by Cabrera et al., although the conclusion was no difference in the ePLR between the groups at different LH levels on the COS initiation day, the ePLRs in the > 2 IU/L and the ≤ 2 IU/L groups were 13.2% and 21.1%, respectively, with trends similar to ours. However, due to their small sample size, the difference using the Chi-squares test was not statistically significant.

In a natural cycle, the serum LH levels remain low in the early stage of follicular development. With the growth of follicles, the level of endogenous LH showed a slight upward trend prior to the LH surge (3). Our results showed a linear correlation in the LH concentrations between the day of COS initiation and the day of the hCG trigger. Further, LH increased slightly due to the development of follicles, which is similar to a natural cycle. Based on the analysis of live births in the fresh ET cycle, the results implied LH on the hCG trigger day might play a key and independent role in the reproductive outcome.

It is still controversial as to whether the LH level after COS affects the outcome of ART. In another agonist study, Westergaard et al. (7) reported that a serum LH concentration < 0.5 IU/L on the 8th day of ovarian stimulation significantly reduced the LBR and increased the ePLR. Lahoud et al. (16) posited that serum LH on D8 was not related to the LBR; however, during the process of COS, a ratio of mid-follicular to early LH concentration of >0.5 could significantly increase the live-birth rate. Even so, Lahoud et al. reported a downward trend in miscarriages as LH increased although without a statistically significant difference. Using an antagonist regimen, Benmachiche et al. (17) discovered a higher live-birth rate and a lower miscarriage rate in patients with a higher serum LH level on the day of the ovulatory trigger. However, Esposito et al. (18) measured the average LH levels from the 5^th^ day of COS onward and found no apparent correlation with the spontaneous abortion rate. A multi-center study also reported no correlation between the LH levels after COS and the ongoing pregnancy rate; however, there was a wide variation in the miscarriage rates in each study center (19).

LH plays a vital role in follicular development (4). Our results demonstrated the same conclusion by showing an association between increasing level of endogenous LH on the initiation day and higher numbers of retrieved oocytes. The endogenous LH did not affect the quality of oocytes because 3 LH levels on the initiation day have no difference in fertilization or high-quality embryo rates. In a study by Ferraretti et al. (11), the addition of exogenous LH to an agonist regimen significantly increased the number of oocytes retrieved, but the fertilization rate remained unchanged. High LH could therefore result in more mature oocytes and more embryos available for ET, with a subsequently increased cumulative live-birth rate (20).

Several studies have shown that LHCGRs are expressed in the lumen and on glandular epithelial cells of the endometrium, as well as on stromal cells (21). LHCGRs likely change periodically with the human menstrual cycle (22). These results suggested that LH might be involved in the developing of the endometrium at the early stages of endometrial proliferation. In our study, endogenous LH on the initiation day of the stimulation cycle only affected the ePLR after fresh ET but showed no correlation with the cumulative ePLR, suggesting that the low levels of LH after suppression might have potential effects on oocyte development and endometrial growth in the stimulation cycle, with the effects being recovered in the subsequent cycle. In summary, in the presence of low LH levels on the initiation day using the agonist regimen, we may choose to delay the time of initiation or perform the COS process after exogenous LH addition to improve the pregnancy outcome.

The limitations of our study included a single center study and its inherited biases from the retrospective design. Only women under the pituitary suppression protocol with a long-acting GnRH-agonist were included in the study. The generalizability of this research to other COS protocols also requires further investigation. Pregnancy loss rates are calculated based on clinical pregnancies and may led to different results depending on the method of calculation. All adjusted ORs are calculated by the model derived from the multivariable logistic regression of existing data. However, there may be other confounders that have not been taken into account.

In summary, although excessive or insufficient suppression of LH may induce luteinization prematurely and seems to be associated with impaired clinical pregnancy rates (23, 24). A low LH level (<1.5 mIU/mL) on the COS initiation day was associated with adverse ART outcomes—including fewer oocytes, higher ePLR and lower LBR in fresh ET—and lower cumulative CPR and LBR in the entire ovarian-stimulation cycle. LH on the COS initiation day was an independent factor affecting ePLR after multivariate regression in fresh ET. Additional studies are needed to characterize the role of LH in early pregnancy loss.

## Data Availability Statement

The original contributions presented in the study are included in the article/supplementary material. Further inquiries can be directed to the corresponding authors.

## Ethics Statement

The studies involving human participants were reviewed and approved by Ethic committee of the First people’s hospital of Yunnan province (KHLL2020-KY013). The patients/participants provided their written informed consent to participate in this study.

## Author Contributions

XL conceived the project and designed the study. XL acquired data and performed statistical analysis. LL, NL, and RM collated the analyzed data. XL wrote the first draft of the manuscript. YL and ZW contributed to data interpretation and provided critical revisions regarding important intellectual content. All authors contributed to the article and approved the submitted version.

## Funding

This work was supported by grants from the Yunnan Provincial Reproductive and Obstetrics and Gynecology Clinical Medicine Center (zx2019-01-01), Open Project of Yunnan Provincial Reproductive and Obstetrics and Gynecology Clinical Medicine Center (2019LCZXKF-SZ01, 2020LCZXKF-SZ06, and 2021LCZXXF-SZ01), National Natural Science Foundation of China (82060282), and Health Science and Technology Plan Projects of Yunnan Province (2018NS0259). The funders had no role in study design; in the collection, analysis, or interpretation of data; in the writing of the manuscript, or in the decision to submit the article for publication.

## Conflict of Interest

The authors declare that the research was conducted in the absence of any commercial or financial relationships that could be construed as a potential conflict of interest.

## Publisher’s Note

All claims expressed in this article are solely those of the authors and do not necessarily represent those of their affiliated organizations, or those of the publisher, the editors and the reviewers. Any product that may be evaluated in this article, or claim that may be made by its manufacturer, is not guaranteed or endorsed by the publisher.
